# Supplementary material for: Nitrogen enrichment stimulates wetland plant responses whereas salt amendments alter sediment microbial communities and biogeochemical responses
Source: PLoS One. 2020 Jul 10;15(7):e0235225. doi: 10.1371/journal.pone.0235225 (PMC7351200; doi:10.1371/journal.pone.0235225)
Supplement: S1 Fig — (a) A mesocosm tank experiment was set up at the University of Connecticut in 2016–2017 to test how plant species and water quality treatments influenced carbon gas fluxes and sediment microbial communities. (b) Co-author O. Johnson monitors real time C fluxes using a transparent floating chamber connected to a Picarro g2201-i during the 2017 growing season. (PDF) [file pone.0235225.s001.pdf]

S1 Fig. **Wetland mesocosm experimental setup.** (a) A mesocosm tank experiment was set up at the University of Connecticut in 2016-2017 to test how plant species and water quality treatments influenced carbon gas fluxes and sediment microbial communities. (b) Co-author O. Johnson monitors real time C fluxes using a transparent floating chamber connected to a Picarro g2201-*i* during the 2017 growing season.

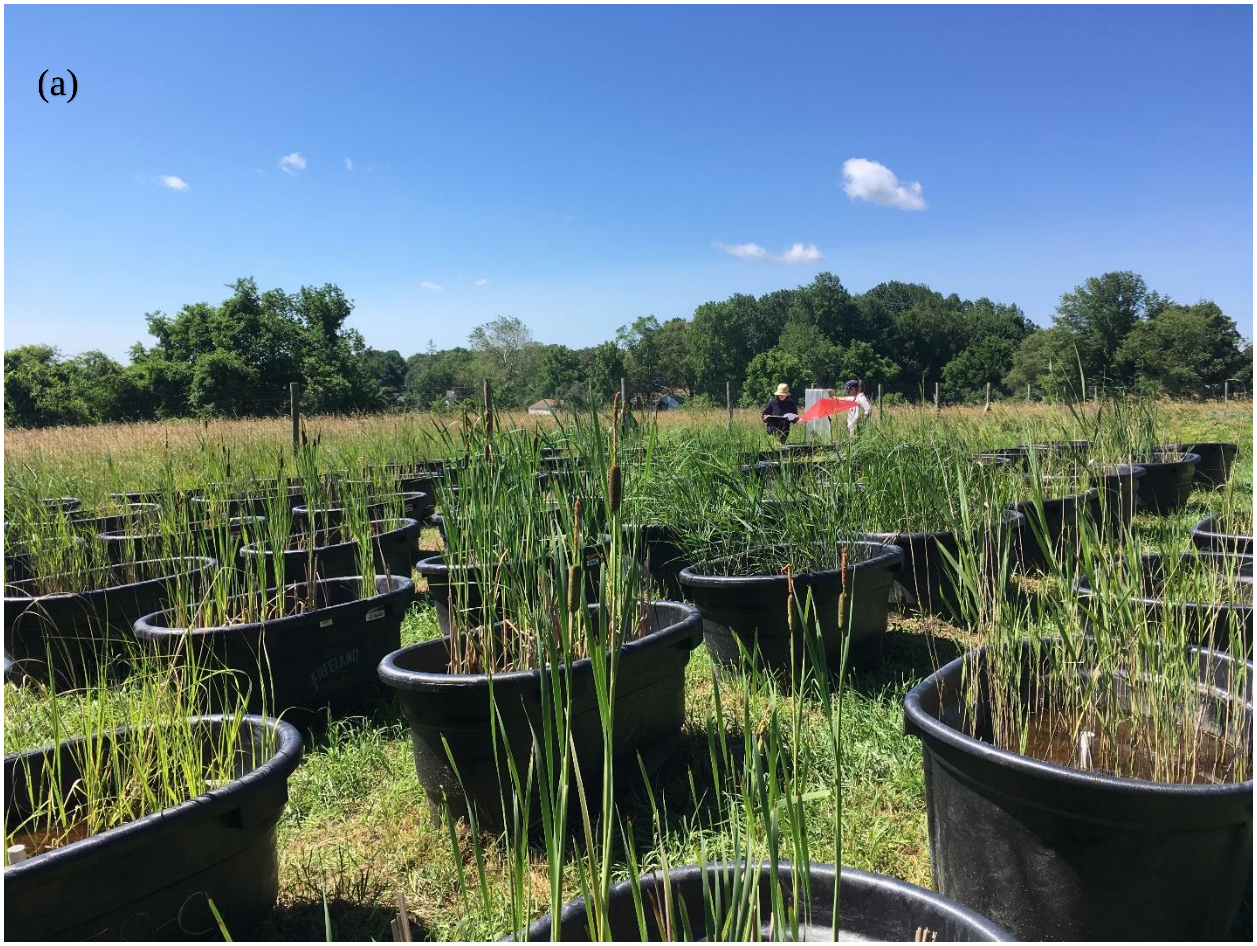

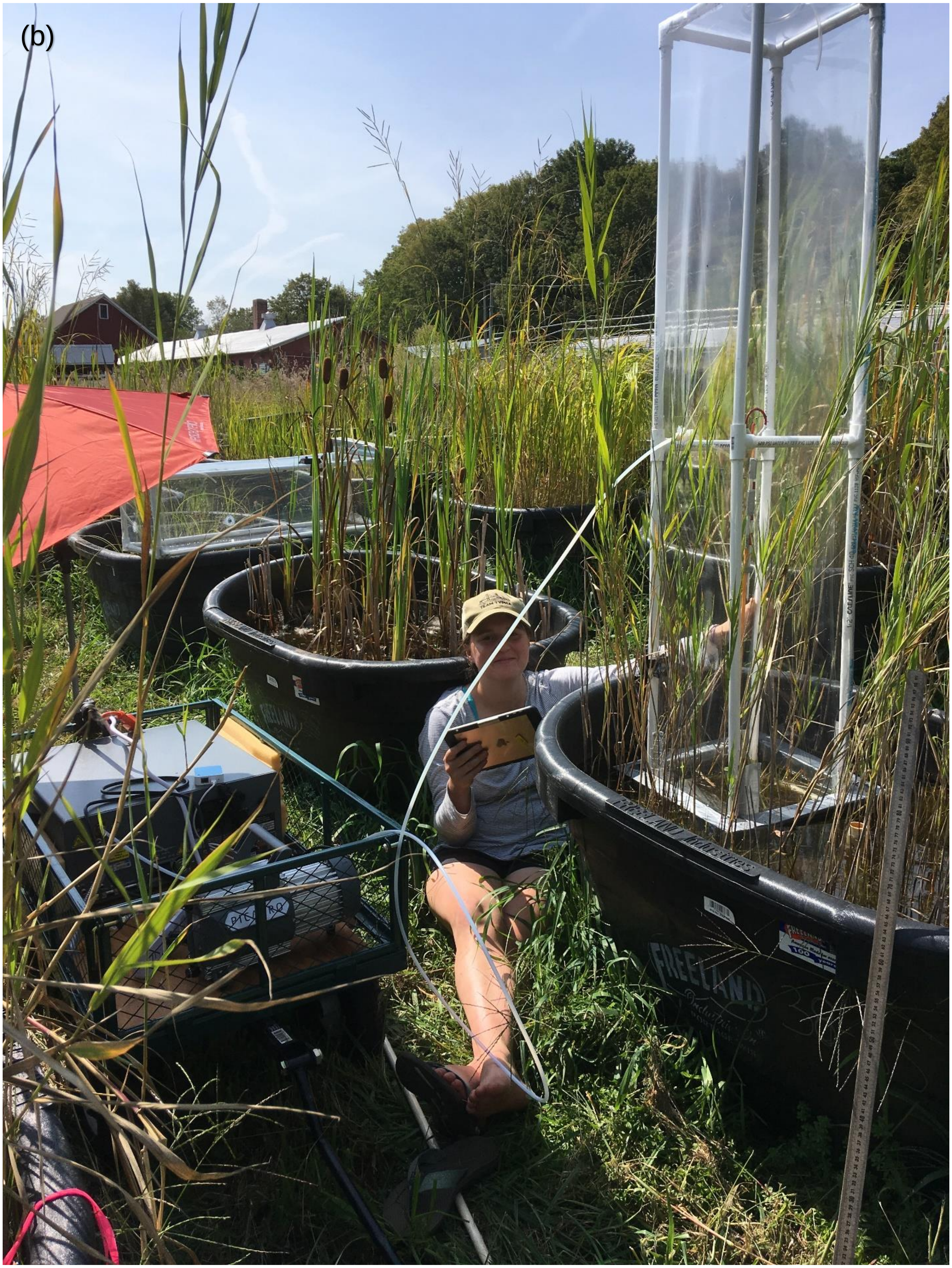

(b)
