# Supplementary material for: Nitrogen enrichment stimulates wetland plant responses whereas salt amendments alter sediment microbial communities and biogeochemical responses
Source: PLoS One. 2020 Jul 10;15(7):e0235225. doi: 10.1371/journal.pone.0235225 (PMC7351200; doi:10.1371/journal.pone.0235225)
Supplement: S1 Table — (PDF) [file pone.0235225.s002.pdf]

S1 Table. **OTUs depleted or enriched in association with the salt treatments.**

OTU	mean control	mean road salt	mean sea salt	Phylum	family
**<u>Enriched in control</u>**					
Otu000026	4.29	0.06	0.00	Proteobacteria	Gallionellaceae
Otu000111	3.76	0.26	0.00	Proteobacteria	Archangiaceae
Otu000048	3.69	0.00	0.00	Proteobacteria	Azospirillaceae
Otu000150	3.11	0.34	1.07	Acidobacteria	Solibacteraceae
Otu000217	2.91	0.46	0.13	Nitrospirae	unclassified
Otu000355	2.82	0.21	0.00	Proteobacteria	Burkholderiaceae
Otu000332	2.56	0.27	0.65	Proteobacteria	Rhodocyclaceae
Otu000552	2.47	0.28	0.72	Proteobacteria	Rhodocyclaceae
Otu000636	2.28	0.00	0.00	Proteobacteria	Magnetococcaceae
Otu001419	1.68	0.00	0.00	Proteobacteria	Acetobacteraceae

**Enriched in salt**					
Otu000017	0.00	4.90	4.97	Verrucomicrobia	Pedosphaeraceae
Otu000031	0.00	4.60	4.87	Bacteroidetes	Microscillaceae
Otu000093	0.00	4.62	3.58	Bacteroidetes	Chitinophagaceae
Otu000515	0.00	2.21	2.82	Proteobacteria	Geobacteraceae
Otu000854	0.00	2.42	1.70	Verrucomicrobia	Pedosphaeraceae
Otu000072	0.03	4.47	4.65	Proteobacteria	Geobacteraceae
Otu000077	0.03	4.47	4.65	Proteobacteria	Betaproteobacteriales_unclassified
Otu000855	0.10	2.22	2.19	Proteobacteria	Caulobacteraceae
Otu000545	0.25	2.82	2.34	Acidobacteria	Solibacteraceae
Otu000307	0.30	3.39	3.03	Acidobacteria	unclassified

Otu000214	0.39	3.97	2.73	Proteobacteria	Nitrosomonadaceae
Otu000235	0.41	3.83	2.68	Proteobacteria	Geobacteraceae
Otu000472	0.46	2.80	2.12	Proteobacteria	Rhodanobacteraceae
Otu000455	0.49	2.99	2.45	Proteobacteria	Xanthobacteraceae
Otu000069	1.12	4.58	4.61	Proteobacteria	Acetobacteraceae
