# Supplementary material for: Nitrogen enrichment stimulates wetland plant responses whereas salt amendments alter sediment microbial communities and biogeochemical responses
Source: PLoS One. 2020 Jul 10;15(7):e0235225. doi: 10.1371/journal.pone.0235225 (PMC7351200; doi:10.1371/journal.pone.0235225)
Supplement: Attachment — Submitted filename: Donato et al_Response to reviews.docx [file pone.0235225.s003.docx]

PONE-D-20-02931 (Response to editor and reviewer comments detailed in blue text below)
Nitrogen enrichment stimulates wetland plant responses whereas salt amendments alter microbial communities and biogeochemical responses
PLOS ONE

Dear Dr. Lawrence,

Thank you for submitting your manuscript to PLOS ONE. After careful consideration, we feel that it has merit but does not fully meet PLOS ONE’s publication criteria as it currently stands. Therefore, we invite you to submit a revised version of the manuscript that addresses the points raised during the review process.

In particular, criteria 3 and 4 ([https://journals.plos.org/plosone/s/criteria-for-publication](https://nam10.safelinks.protection.outlook.com/?url=https%3A%2F%2Fjournals.plos.org%2Fplosone%2Fs%2Fcriteria-for-publication&data=02%7C01%7Cbeth.lawrence%40uconn.edu%7C1d3cfd11ff6048e6df1908d7beeb1209%7C17f1a87e2a254eaab9df9d439034b080%7C0%7C1%7C637187789146038160&sdata=ovQnbTQY2DExobcfp%2BF2xzgZOhoqLAYIRaosF48Z3so%3D&reserved=0)) have not been fully achieved. Experimental and statistical issues were raised by both reviewers, which need to be addressed by the authors as well as it is crucial to verify the values of tables/figures and text to avoid any misleading information.

Response: We thank the reviewers and editor for their thoughtful recommendations and have addressed all statistical issues raised and have also verified the values in all tables and figures. Please see our detailed responses below.

**I also have few additional points:**
- Have the authors considered the distinction between heterotrophs/organotrophs and heterotrophs/lithotrophs, when presenting the results in lines 416-419? These distinctions, in combination with data on salt tolerance could bring more light further into the discussion. These characteristics should then be included in table S1 and should also be used in the discussion of the results (lines 482-503).

Response: We have purposefully avoided too much discussion of potential functions of the differentially abundant OTUs for several reasons. First, we do not have cultures for the organisms so the identification is based on a single gene fragment, thus any identifications have to be taken with a grain of salt. To highlight this we have added the genus level classification to Table S1, which shows that many (12 of 13) OTUs could not be reliably classified. Second, changes in abundance are not always associated with a change in activity. So seeing an increase or decrease in an OTUs abundance does not mean any function assigned to that OTU would manifest as a net change in that activity in the community. Finally, many organisms show mixotrophic growth showing organo/lithotrophic characteristics depending on environmental conditions. So it is not always a simple matter to assign these functions.

That being said, we agree that there may be more information available than we presented and have added the following to the results (Lines 433-447): “For instance, an OTU related to the genus Sideroxydans, an iron oxidizing group of bacteria (Muhling et al, 2016), was enriched in the control samples (Table S1). In contrast, three OTUs related to the genus Geobacter were enriched in the salted sediments (shared in both road salt and sea salt). Members of the Geobacter genus are thought to be the primary drivers of oxidizing organic matter coupled to the reduction of iron and manganese (Lovley et al., 2011). In this respect, these data point to a state change in the iron cycle in the mesocosms under the salt treatments, which points to a decreased availability of dissolved iron under elevated salt. The remaining OTUs largely belonged to general heterotrophic bacteria or were not able to be classified to taxonomic ranks deeper than family, which limits the confidence that functional predictions can be made from these classifications.”

and the discussion (Lines 513-527): “The differentially abundant OTUs did point to an alteration in the iron cycle in the sediments under the salt treatment. The enrichment of Sideroxydans in the control mesocosms in comparison to an enrichment of Geobacter with elevated salt suggests a shift from iron oxidation to iron reduction with the addition of salt. Further, similar to previous experimental findings [Craig and Zhu 2018], we observed reduced mineralization of labile carbon from the salt treatments, which may have been associated with reduced microbial diversity or shifts in community composition. Thus, the osmotic or redox stress induced by the salt treatment did appear to shift biogeochemical cycles in the sediments.”

- The authors in the material and methods section clearly indicated that bacterial communities were not collected from the rhizosphere, but from bulk soil from the root zone. I believe the discussion in lines 473-481 would be improved if the authors also related the results with known effect of soil properties/soil parent material in the bacterial communities.

Response: We have added the following to the section (Lines: 420-426): “Taken together, these data suggest that the differing plant responses to the N, road salt, and sea salt treatments were not matched by similar responses in the sediment microbial community. Given that the soils for this survey were collected in the vicinity of the roots, but did not include the rhizosphere soils directly in contact with the roots, the influence of the plant on sediment communities did not appear to extend into the root zone soils. Instead, sediment microbial communities appeared to respond to changes in sediment properties, particularly those associated with salting, such as osmotic stress.”

**Some minor points:**

Line 384-388, besides, the genus of the identified OTUs, also indicate in brackets the phylum;

Response: The suggested changes have been made.

Figure 8, in particular in Figure 8a, please change (Average counts treatment (log10)) by (ratio of average counts treatment (log10), controls versus treatment);

Response: The suggested changes have been made.

Line 456 replace “root porocity” by “root porosity”.

Response: This change has been made.

Journal Requirements:

When submitting your revision, we need you to address these additional requirements.

1. Please ensure that your manuscript meets PLOS ONE's style requirements, including those for file naming. The PLOS ONE style templates can be found at

[http://www.journals.plos.org/plosone/s/file?id=wjVg/PLOSOne_formatting_sample_main_body.pdf](https://nam10.safelinks.protection.outlook.com/?url=http%3A%2F%2Fwww.journals.plos.org%2Fplosone%2Fs%2Ffile%3Fid%3DwjVg%2FPLOSOne_formatting_sample_main_body.pdf&data=02%7C01%7Cbeth.lawrence%40uconn.edu%7C633638a0f2ed4609785808d7e11eb70d%7C17f1a87e2a254eaab9df9d439034b080%7C0%7C1%7C637225394358522972&sdata=RFaLuS%2FMvgK4JKWN0bEELDusAWLZYac%2BH6D66%2FBUgNI%3D&reserved=0) and [http://www.journals.plos.org/plosone/s/file?id=ba62/PLOSOne_formatting_sample_title_authors_affiliations.pdf](https://nam10.safelinks.protection.outlook.com/?url=http%3A%2F%2Fwww.journals.plos.org%2Fplosone%2Fs%2Ffile%3Fid%3Dba62%2FPLOSOne_formatting_sample_title_authors_affiliations.pdf&data=02%7C01%7Cbeth.lawrence%40uconn.edu%7C633638a0f2ed4609785808d7e11eb70d%7C17f1a87e2a254eaab9df9d439034b080%7C0%7C1%7C637225394358522972&sdata=i2HECcD0RGPlAE8VFG%2BhT6nwSOWw3IP%2Bn4g%2FX9r9fto%3D&reserved=0)

Response: We now conform to PLOS ONE’s style requirements.

2. We note that you have stated that you will provide repository information for your data at acceptance. Should your manuscript be accepted for publication, we will hold it until you provide the relevant accession numbers or DOIs necessary to access your data. If you wish to make changes to your Data Availability statement, please describe these changes in your cover letter and we will update your Data Availability statement to reflect the information you provide.

Response: All our raw sequence data are available in the NCBI Short Read Archive (SRA) under the BioProject ID PRJNA604015: https://www.ncbi.nlm.nih.gov/bioproject/?term=PRJNA604015. All other data will be available after acceptance in the Dryad database.

3. Please amend either the title on the online submission form (via Edit Submission) or the title in the manuscript so that they are identical.

Response: Titles are now identical.

4. Please include captions for your Supporting Information files at the end of your manuscript, and update any in-text citations to match accordingly. Please see our Supporting Information guidelines for more information: [http://journals.plos.org/plosone/s/supporting-information](https://nam10.safelinks.protection.outlook.com/?url=http%3A%2F%2Fjournals.plos.org%2Fplosone%2Fs%2Fsupporting-information&data=02%7C01%7Cbeth.lawrence%40uconn.edu%7C633638a0f2ed4609785808d7e11eb70d%7C17f1a87e2a254eaab9df9d439034b080%7C0%7C1%7C637225394358532967&sdata=7%2FcYXXEMlqY%2BCiuBGkatKhPFMD76fmKTl4EZBODkzIQ%3D&reserved=0)

Response: Captions for Supporting Information files are now included at end of the manuscript according to guidelines provided.

5. Our internal editors have looked over your manuscript and determined that it is within the scope of our Call for Papers on the Microbial Ecology of Changing Environments. Additional information can be found on our announcement page: [https://collections.plos.org/s/microbial-ecology](https://nam10.safelinks.protection.outlook.com/?url=https%3A%2F%2Fcollections.plos.org%2Fs%2Fmicrobial-ecology&data=02%7C01%7Cbeth.lawrence%40uconn.edu%7C633638a0f2ed4609785808d7e11eb70d%7C17f1a87e2a254eaab9df9d439034b080%7C0%7C1%7C637225394358532967&sdata=JyQmI1nTC16qPtssGD%2FFPZZbVViYQkwjCwUzGLuCTVI%3D&reserved=0). If you would like your manuscript to be considered for this collection, please let us know in your cover letter and we will ensure that your paper is treated as if you were responding to this call. If you would prefer to remove your manuscript from collection consideration, please specify this in the cover letter.

 Response: Great- this sounds like a good opportunity and I will indicate our interest in being included in our cover letter if it is still feasible given our resubmission is beyond the April 10^th^ deadline.

6. In your Methods section, please provide additional information regarding the permits you obtained for the work. Please ensure you have included the full name of the authority that approved the field site access and, if no permits were required, a brief statement explaining why.

 Response: The experiment was conducted on property owned by the University of Connecticut, where I am a faculty member, thus, no permits were required.

**Reviewers' comments:**

**Reviewer #1:** Here Donato et al. analyzed the impacts of salt and nitrogen deposition on carbon cycling in wetland monocultures. They used 48, ~380 L tanks to make 4 replicates of 3 different species with 4 different water chemistry treatments. Overall the authors make a strong case for how salts impact both vegetation and microbial communities and thus the carbon cycling in wetlands. The study is original but some work needs could be done to contextualize the results within the broader literature to help expand the relevance of the mesocosm study the authors present (see papers below). This is particularly true when discussing the relevance of the OTU abundance on carbon cycling. Salt amendments lowered C mineralization, and altered OTU diversity, but the link between the two needs to be more carefully addressed within the discussion. Finally, the authors need to address the design components which could have impacted the conclusions.

Response: Please see our response to the editor above related to linking OTU abundance and function. We amended our discussion to address how OTU diversity may alter sulfur, iron, and carbon mineralization (Lines 433-447), and now reads “For instance, an OTU related to the genus *Sideroxydans*, an iron oxidizing group of bacteria [57], was enriched in the control samples (Table S1). In contrast, three OTUs related to the genus *Geobacter* were enriched in the salted sediments (shared in both road salt and sea salt). Members of the *Geobacter* genus are thought to be the primary drivers of oxidizing organic matter coupled to the reduction of iron and manganese [58]. In this respect, these data point to a state change in the iron cycle in the mesocosms under the salt treatments, which points to a decreased availability of dissolved iron under elevated salt. The remaining OTUs largely belonged to general heterotrophic bacteria or were not able to be classified to taxonomic ranks deeper than family, which limits the confidence that functional predictions can be made from these classifications.”

It is unclear if the authors consider the impacts of draining the tanks on the reduction-oxidation potential of the micropores within the soil? Although the seasonal methane signal was not described in the text, the relatively large p-value suggests that methane may not have been consistent across all sampling periods. Could exposure of the soil matrix to the atmosphere be part of the story? The authors should address this.

Response: As we describe, “initial repeated measures ANOVA (lme command) indicated consistent treatment responses across our three sampling campaigns for both CO2 (F2,94 = 0.83, p = 0.439) and CH4 fluxes (F2,87 = 2.64, p = 0.077), thus we aggregated gas flux data into one data set for statistical analyses of gas flux response.” It is unclear what you mean by the “relatively large p-value”; did you intend to say relatively *low* p-value? We now consistently use an alpha level of 0.05 to infer statistical significance, so we stand by our decision to aggregate fluxes across sampling campaigns. However, it is certainly possible that there was an ecologically relevant temporal trend in CH4 fluxes; this could be related to how we manipulated hydrology, temperature, plant inputs, or their combination. We drained the tanks at the end of the growing season soon before the first freezing temperatures, and re-filled them as temperatures started to warm up; thus the tanks were filled with water when temperatures were prime for microbial activity and any oxygen dissolved in pore water was likely quickly consumed. We now address this and other possible issues associated with our experimental design in the discussion, in a section titled “Experimental design constraints” (Lines 546-559).

The Gibbs free energy that determines which terminal electron acceptor is the dominant metabolic pathway is pH dependent (See Bethke et al. 2011). If the soils and treatments varied in acidity, particularly around neutral conditions, metal reduction rates could be more relevant than the SO4 content in the context of limiting methanogenesis. If the pH wasn’t measured, then the authors need convince the reader that the pH is similar across all treatments or they need to soften their language around ‘favorablitily’ of the kinetics that regulate microbial metabolisms.

Response: This is a great point and we soften our language about the kinetics of microbial metabolism as recommended, as others have found reduced pH with road salt addition (Craig and Zhu 2018, Kim and Koretsky 2013). Unfortunately, we did not measure soil pH during our study, but the water we used to maintain water levels was circum-neutral (pH of 7.18).

The statistics reported throughout the paper need some improvement. In one case the authors accept a null hypothesis with a p-value of 0.077 (line 222), but then reject it in separate analyses with a p-value of 0.078 (Ln 267) and 0.069 (Ln 269). Liner mixed effect models were poorly described but used to justify aggregating methane fluxes despite relatively low p-values. This may be acceptable, or may be problematic depending on the specific hierarchal model design.

Response: We apologize for the inconsistency. We re-wrote the results so that responses with p > 0.05 are not considered statistically significant. The sentence now reads (Lines: 270-274), “Biomass production differed among vegetation above- and belowground (above: F2,36 = 46.5, p < 0.001; below: F2,36 = 6.8, p = 0.003) and among water quality treatments aboveground (above: F3,36 = 144.0, p < 0.001), but we observed interactions between species and water quality treatment for aboveground biomass (F6,36 = 6.5, p < 0.001), principally because Spartina aboveground biomass responded strongly to N enrichment (Fig 1).”

The correlation analyses could also use some clarification, since it was unclear when Pearson correlation were used or when the Spearmen rank test was implemented. Clarity would help the reader understand the decisions around test selection, as it was initially assumed by this reviewer that the log transform was done to normalize the data. Why then use a Spearmen rank test in this instance, or if using Spearmen, why log transform the data?

Response: We changed the text to clarify why we used Pearson vs. Spearman’s tests. The text now reads (Lines: 229-233): “We used Pearson’s correlation coefficient (r) for parametric data and Spearman rank correlation coefficients (r_s_) for non-parametric data (i.e., when transformations did not improve normality).” We clearly indicate in the results what test we used for each pair of variables by using the r or r_s_ notation.

Furthermore, regression analysis may be more informative than Pearson in a number of analyses presented here, since it allows for multivariate analysis and post hoc analysis of residuals. For example, a properly constructed linear model could determine if the correlation between SO4 and methane is independent of the Spartina group, which can not be inferred from the Person correlation analysis presented in the text.

Response: If possible, we agree a multivariate analysis would have been more informative, however we did not have sufficient sample size to run multivariate analyses (i.e., we used gas flux data from a single sampling campaign, so total n = 48, divided by 4 water quality treatments = 12, divided by 3 plant species = 4 reps per group for multivariate analysis).

Recommended Papers:
Granberg, G., Sundh, I., Svensson, H., and Nilsson, M., Effects of temperature and nitrogen and sulfur deposition on methane emission from a boreal mire, Ecology, 82, 1982–1998, 2001.
Bethke, C. M., Sanford, R. A., Kirk, M. F., Jin, Q., and Flynn, T. M., The thermodynamic ladder in geomicrobiology, Am. J. Sci., 311, 183–210, 2011.
Herndon, E. M., Mann, B. F., Roy Chowdhury, T., Yang, Z., Wullschleger, S. D., Graham, D., Liang, L., and Gu, B., Pathways of anaerobic organic matter decomposition in tundra soils from Barrow, Alaska, J. Geophys. Res.-Biogeo., 120, 2345– 2359, 2015.
Christiansen, J. R., Levy-Booth, D. J., Prescott, C. E., and Grayston, S. J., Microbial and environmental controls of methane fluxes along a soil moisture gradient in a Pacific coastal temperate rainforest, Ecosystems, 19, 1255–1270, 2016.
Gao, C., Sander, M., Agethen, S., and Knorr, K.-H., Electron accepting capacity of dissolved and particulate organic matter control CO2 and CH4 formation in peat soils, Geochim. Cosmochim. Ac., 245, 266–277, 2019.
Clark, M. G., Humphreys, E. R., Carey, S. K., Low methane emissions from a boreal wetland constructed on oil sand mine tailings, Biogeosciences, 17, 667-682, 2020.

Response: Thank you for the literature recommendations. We incorporated considerations from Gao et al. 2019 and Bethke et al. 2011 into the discussion.

**Specific comments:**
Line 84: Maybe some reclamation lit here? Response: We assume you are referring to mine reclamation, but that is unclear. Since our study focuses on the effects of common wetland pollutants of eastern North America (nitrogen, road and sea salt), we do not think it is necessary to integrate reclamation literature here.
Line 53: Are they highly productive, or do they just have low rates of respiration? The 5th assessment report WG1 is not a good source for suggesting that wetlands are becoming more monotypic graminoid dominant. Response: You are correct, this is not an appropriate citation. We now cite Zedler and Kercher 2004, which is the citation we were intending to use originally. We checked accuracy of all citations throughout manuscript.

Line 143: A schematic, of field picture to help visualize the experimental design would be helpful. Even if it’s in the supplementary data.
Response: Thank you for the suggestion. We have added Supplemental Figure 1 which includes two photos: (a) our experimental mesocosm set up and (b) our gas sampling chamber configuration.

Line 161: What is the accuracy on the allometric equations?
Response: We added the R^2^ and 95% Confidence Intervals for our allometric equations.

Line 273: Imprecise wording. I think it means it is relative to the control and the salt treatments? The control isn’t really a “treatment”.
Response: We clarified wording here. It now reads, “Nitrogen enrichment increased biomass and reduced fPAR (N-enrichment: 52.9% ± 2.9) relative to the average of the control and salt treatments (72.5% ± 1.1).”

Lines 290/91: This sentence is confusing. Do you simply mean there was a species and treatment effect but no interaction term? Simple wording clarification should be fine.
Response: We clarified wording here. It now reads, “We observed differences in CO2 uptake among species (F2,36 = 34.27 p < 0.0001) and among water quality treatments (F3,36 = 12.48, p < 0.0001), but did not observe an interactive effect of species and water quality treatment (F6,36 = 1.12, p = 0.369).”

Line 294/95: No mention the road salt treatment.
Response: We added road salt treatment result. It now reads, “Nitrogen addition (39,564 ± 3467 µmol m^-2^ h^-1^) increased CO_2_ uptake relative to freshwater controls (19,392 ± 1618 µmol m^-2^ h^-1^) and sea salt treatments (19,751 ± 1990 µmol m^-2^ h^-1^), but did not lead to different CO_2_ uptake from our road salt treatment (20,211 ± 1467 µmol m^-2^ h^-1^; Fig 2b).”

Line 304: There was an issue with the µ (/mu) symbol on the pdf.
Response: All gas flux rates are in units of µmol m^-2^ h^-1^. Figures 2 and 3 are on a log scale. We checked the units to confirm they are accurate.

Line 441: ends with a citation, but is written as if it was a result from this study.
Response: Yes, we clarified that this line was referring to Ref #46. It now reads, “Dramatic biomass reductions for freshwater macrophytes were observed when salinity treatments exceeded 4 ppt in [46].”

Lines 429-430 is vague wording. Is it largely similar in response to water quality treatment or largely similar in the differences across species within each treatment?
Response: We clarified wording here. It now reads, “We found that plant species had strong effects on our response metrics, with largely similar patterns in response to water quality treatments across plant species.”

Line 438-40: “environmentally relevant” and “we targeted” is redundant here. You already explained in the methods that you targeted environmentally relevant salinity profiles. Response: We felt it was appropriate to comment on the environmental relevance in the discussion as well.

Line 440-442: Did you mean “Other studies have found…”? Response: Yes, we clarified that this line was referring to Ref #46. It now reads, “Dramatic biomass reductions for freshwater macrophytes were observed when salinity treatments exceeded 4 ppt **in** [46].”

Line 442-443: Did Walker S. (2019) find only an effect at >2 ppt? Your wording leaves their results ambiguous. Did they find no effect at <2 or didn’t include it in their study? Response: We clarified the text here so that it was clear that Walker et al. tested a range of salinities. It now reads, “Likewise, [39] observed a wetland seed bank threshold of 2 ppt for species richness, diversity, and aboveground biomass, with reductions in plant responses in NaCl treatments > 2 ppt, suggesting that common freshwater wetland plants may be resilient to salinity levels ≤ 2 ppt.”

Line 447: “across all treatment” sounds like correlation analysis was performed within each treatment but was not reported. Response: We added clarification in the statistical analyses section that the correlation was for entire data set, not by treatment.

Lines 450-452: The correlation to DOC is a separate idea. Put it in a new sentence for clarity unless it was only negatively correlated in the salt treatments. Response: We made the suggested edit.

Line 453: Was the relationship between microbial community abundance and heterotrophic respiration examined, why wasn’t it reported? I would assume some of these communities would have no impact on aerobic respiration. Response: We did not directly examine the relationship between microbial community and heterotrophic respiration as it is difficult to link community changes to flux rates given our methodology (see above response to editor). We see an overall decrease in diversity of the community which could indicate lower functional diversity. However, the elevated respiration with nitrogen addition was not associated with a change in the microbial communities. We added these ideas to the discussion (Lines: 478-483).

Line 457: reference reads as if its on your own findings. Perhaps add “…as others [ref] have described”?
The paragraph starting on Line 455 is difficult to follow. A lot of ideas that jump around and don’t build on one another. Reorder the SO4-Spartina discussion so it builds to the conclusion, that methane is largest in these plots. The second sentence states that “Our data shows” but has two citations. You need to say what specifically in the other studies relate to what your data shows. Response: We clarified wording as suggested. It now reads, “Still, our data suggest that porous plant tissue acted similarly to a straw, allowing methane produced in anoxic sediment to bypass surface oxic layers and travel into the atmosphere, as observed by other studies [13,59].”

Lines 468/69: Why “this is surprising” is unclear, since expectations for production/emission by species was never discussed. Is the surprise simply referring to the productivity, the methane flux, or both?
Response: We agree this was unclear, we removed “why this is surprising” and focused on the main idea, that “This is likely associated with the seed source we used…”

Line 483: Is this in reference to Fig 6b, not 4b? Response: Yes, we corrected figure reference.

Line 487: Again, I think this is Fig 8C. Fig 6A has nothing to do with rarity of populations. This sort of repeated mistake makes it hard to follow the discussion and has me wonder what else has been overlooked. Response: We corrected the figure reference and confirmed accurate figure numbers throughout the manuscript.

Line 492: I am not a microbiologists. For those who share this shortcoming, I wonder if you could add the primary metabolic pathway (i.e. sulphate reducers, methanogens, etc.) of the microbes to table S1 or perhaps list it when you first discuss the Phyla of the microbes? It would help readers follow the logic in the discussions on species abundances and reinforce the connection to the carbon cycle.

Response: Please see our response to the editor above. To reiterate briefly, it is difficult to assign a function to a microbe that we have only identified from a single gene fragment in a sequence-based assay. We have expanded the results and discussion to encompass some of the functional predictions we can make. Please see Lines 433-442, and 513-520.

Line 503: Did you look into composition/abundance and mineralization rates? I didn’t see it in your manuscript but could be another link between microbes and the carbon cycle.
Response: See our response to previous comment and in our response to the editor.

**Discussion:** What are the implications of reduced fPAR on the carbon cycle with respect to your treatments? It was not included in the discussion, so is it relevant to the study?

Response: We included the potential importance of fPAR in the discussion (Lines 466-469), and the relevant next reads “Anecdotally, we observed higher algae abundance in surface waters of *Typha* and *Phalaris* with N addition; higher levels of PAR penetrating through sparser canopies may have stimulated algal production and resulted in similar increases in CO_2_ uptake across all vegetation treatments with N addition.”

Readability of Figure 8 is very low. Impossible to see axis labels. Increase resolution. Response: We revised Fig 8 to increase the readability as recommended.

**Reviewer #2:** Donato and collaborators are describing the carbon fluxes and microbial changes in wetland mesocosms exposed to nitrogen, road salt, or sea salt contaminations. They showed that carbon fluxes were mainly impaired by Nitrogen treatment through plant biomass changes, the salt pollution disturbed C mineralization, decrease in microbial diversity and shifts in the microbial community, and led to lower CH4 emissions.The authors have made a great effort in compiling a complex dataset in a very comprehensive study. The study is detailed, and the results well explained. The conclusions are interesting and will be of interest to the PLOS ONE readers. My main comments would be that the authors do not discuss the eutrophication of their system, neither the (likely high) reducing environment that the N and salt treatment likely generated. Though an additional discussion of that matter would be interesting, it should not prevent the publication of this paper that is already convincing as it is.

See my other comments below:
1. Was anything added to the control treatment (eg 1L of DIW?) Response: Yes, 1L of DI W was added to control treatments. We added this detail to the text.
2. Line 162: Please provide a reference or in SI the correlations used to calculate the plant biomass based on their height. Response: We added the R^2^ and 95% Confidence Intervals for the equations.
3. It would be of interest to specify that the sampling campaigns were done 1months, 2 months, and 3 months after the last dosing treatment, during the summer (right?) Response: We clarified this so that the text now reads, “We measured C fluxes during three sampling campaigns in 2017 (mid-July, August, and September; approximately one, two, and three months after the last dosing treatment).”
4. Line 286: significances between treatments or between plant species? Line 280 = treatments do not impact root porosity) Response: Current Lines 284-286 are referring to our observation of no differences among water quality treatments, whereas Table 1 presents differences among plant species.
5. All plot, I would call the panels “treatments” rather than “Water quality”, which is a trait itself. It is a bit confusing as it. Response: We manipulated vegetation and water quality, so consider them both “treatments.” To avoid potential confusion, we changed the panel headers on Figures 2, 3, and 4 from “Water quality” to “Water quality treatments.”
6. Line 303 and fig 3a: the number provided in the text vs the values plotted doesn’t seem to match. Response: The values in Fig 3 are log-transformed data. We provided untransformed data in the text to allow easy comparison with other studies.
7. It has been shown in similar systems that redox variations can vary daily and along the year1, and be impacted by anthropogenic activities. These effects were likely mainly driven by the macrophyte photosynthesis, respiration, and life cycles. I am pointing this because based on my (rough) calculations, the authors have added approximately 5g of SO42- in their system (based on the characterization provided in ref 41 in the manuscript), which should represent a concentration in the 20L mesocosms of 250 mg/L. Despite the very high addition of SO42- added to their system the authors measured “only” 3.2 mg SO42- per L of water in their mesocosms. This likely indicates a reducing environment (which is not surprising since the sampling was done during the summer, as described in ref1).
My question is: could it be that some of the observed effects in the sea salt treatment (decrease of DOC, C mineralization) are due to a more reducing environment than the others? Did the authors measure dissolve CO2 and O2 concentrations in the mesocosms water? Response: We did not measure dissolved CO_2_ or O_2_ concentrations in mesocosm porewater. Since wetlands are characterized by reducing conditions, we intentionally manipulated the hydrology to promote anoxic, reducing conditions. We assume oxygen was consumed equally in all water-logged soils, leaving the explanations for observed effects to be related to terminal electron acceptor availability, and salt effects on organic matter and osmotic pressure. Sea salt treatments still had twice as much SO42- than any other treatment, indicating more SO42- available for sulfate reducers, and we infer the decreased DOC is due to increased flocculation of organic matter due to salt addition, and the decrease in C mineralization is due to osmotic stress on microbes.

8 Line 485: it could be osmotic stress and/or a more reducing environment.

Response: We have added redox stress to the section. Thank you for the suggestion.

9 Line 463: Spartina is also very responsive to the nitrogen treatment. Could it be an indicator of the eutrophication of the system, thus more reduction of the water, explaining the larger decrease in SO42- concentration? I am surprised that the authors do not discuss eutrophication here when implementing their systems with N at a very high rate (15 g N /year)

Response: It is possible that *Spartina* has higher nutrient use efficiency than the other species, as *Spartina* increased aboveground biomass 5x more than controls, compared to 3x increase of *Typha* and *Phragmites* relative to controls. Excess N and higher light penetration may have stimulated algal growth in surface waters, which we anecdotally observed in *Typha* and *Phragmites* tanks. Interestingly, we did not observe an effect of N addition on C mineralization rates, indicating excess nutrients were assimilated by plants, algae or microbes in water column, but not microbes in soil. We added these ideas to our discussion (Lines 463-469).

10 Line 491:Figure 5 also indicates that the 2salts treatments induce a shift toward the CCA1 factor, and the 2 populations are grouped. This could indicate that, at that period of the year (already under pretty high reducing conditions), the microorganisms are more sensitive to high salinity than to the redox conditions. Response: Good point- we amended text to read “We observed a reduction of methane emissions in the sea salt treatment, yet we did not observe an enrichment of sulfate reducers (S1 Table) which could indicate higher sensitivity to salinity than to redox conditions”

11 I could be of interrest for the reader to add a picture of the mesocosms in supporting information Response: We added a photo of both our experimental design and our gas sampling chamber set-up to the SI as recommended.

Cited reference
1 Andersen, et al. "Extreme diel dissolved oxygen and carbon cycles in shallow vegetated lakes." Proceedings of the Royal Society B: Biological Sciences 284.1862 (2017): 20171427.
2 Simonin, et al. "Engineered nanoparticles interact with nutrients to intensify eutrophication in a wetland ecosystem experiment." Ecological Applications 28.6 (2018): 1435-1449.

Response: Thank you for the suggested references. We integrated Simonin et al. 2018 into the discussion, and highlight the need to further investigate emerging contaminants in wetlands.

While revising your submission, please upload your figure files to the Preflight Analysis and Conversion Engine (PACE) digital diagnostic tool, [https://pacev2.apexcovantage.com/](https://nam10.safelinks.protection.outlook.com/?url=https%3A%2F%2Fpacev2.apexcovantage.com%2F&data=02%7C01%7Cbeth.lawrence%40uconn.edu%7C1d3cfd11ff6048e6df1908d7beeb1209%7C17f1a87e2a254eaab9df9d439034b080%7C0%7C1%7C637187789146068143&sdata=gNFyOlQvPsO61ohzC6v%2B0BFO9dEeYlfogTv2YdyFU30%3D&reserved=0). PACE helps ensure that figures meet PLOS requirements. To use PACE, you must first register as a user. Registration is free. Then, login and navigate to the UPLOAD tab, where you will find detailed instructions on how to use the tool. If you encounter any issues or have any questions when using PACE, please email us at [figures@plos.org](mailto:figures@plos.org). Please note that Supporting Information files do not need this step.

Response: All our figures (including revised versions) meet PLOS requirements, as determined by uploading to PACE.
