# Supplementary material for: Nitrogen enrichment stimulates wetland plant responses whereas salt amendments alter sediment microbial communities and biogeochemical responses
Source: PLoS One. 2020 Jul 10;15(7):e0235225. doi: 10.1371/journal.pone.0235225 (PMC7351200; doi:10.1371/journal.pone.0235225)
Supplement: Attachment — Submitted filename: Donato et al_6.4.20_response to reviews.docx [file pone.0235225.s004.docx]

PONE-D-20-02931R1 (Response to editor and reviewer comments detailed in blue text below)

Nitrogen enrichment stimulates wetland plant responses whereas salt amendments alter sediment microbial communities and biogeochemical responses
PLOS ONE

Dear Dr. Lawrence,

Thank you for submitting your manuscript to PLOS ONE. After careful consideration, we feel that it has merit but does not fully meet PLOS ONE’s publication criteria as it currently stands. Therefore, we invite you to submit a revised version of the manuscript that addresses the points raised during the review process.

The authors addressed most of the previous reviewer’s concerns, however some minor points need to be addressed. Please modify the manuscript according to reviewers’ suggestions. Moreover, use the same format for units all over the manuscript (including figures) (mg/L or μmol m^-2^ h^-1^).

Response: We have addressed all reviewer concerns (detailed below) and confirmed the consistent use of units throughout the manuscript. Note: Fig 4 illustrates differences in C mineralization among vegetation and water quality treatments, which is expressed as a rate per mass of soil, not on an aerial basis, so units are different from other C fluxes in Figs 2 and 3.

Reviewer #1: This version is much stronger submission. Donato et al. clearly structure and organize their explanation of the impacts of nitrogen and salt treatments on wetland mesocosm’s. The results, attempting to bridge fields in biogeochemistry, botany, ecology, and microbiology are relevant and topical. The discussion could be extended to wider literature, but I do not believe that should prevent the publication of this manuscript. I also appreciate that the limitations are now clearly addressed in their own section within the manuscript. I believe this manuscript would be of interest to the readers of PLOS ONE. I have a few very minor comments below.
General:
- Some figures have the first sentence of the caption bolded, some do not (Figures 3 & 5). Response- All figures and tables now have their title bolded.
- Microbial community response results are clearly tied to the rest of the manuscript in this draft, but as currently written some of the “results” would be better suited for the discussion section. Response: Good point. We moved and altered some of the text (former Lines 422-428) from the microbial results section into the discussion.

Specifics:
Line 222: Clearly state what the random effects are, I have assumed individual gas flux plot location (i.e. repeated measures). Response: Changed as recommended. Text now reads “We tested for fixed effects of plant species, water quality treatments, and their interaction on gas fluxes, biomass, total porosity, and water chemistry data using Linear Mixed Effects models (*lme* and *aov* commands), with mesocosm tanks set as a random effect. Initial analysis (*lme* command) indicated consistent treatment responses across our three sampling campaigns for both CO_2_ (F_2,94_ = 0.83, p = 0.439) and CH_4_ fluxes (F_2,87_ = 2.64, p = 0.077), thus we aggregated carbon flux data from the three sampling campaigns to test for differences among vegetation and water quality treatments.”

Line 224: lme stands for Liner Mixed-Effect Model, although an LME is an acceptable way to construct a repeated measures ANOVA, I suggest that since you started with the mixed effect language above (Line 222) you should stick to one terminology for clarity. Response: Good point. We changed as recommended. See altered text in response to comment above.

Line 227 states that “only the September gas sampling data is used” when investigating relationship with other “variables” and Line 229/30 states “tested for correlations across the entire data set”. This language is imprecise. I believe all 3 gas sampling campaigns were used for carbon flux analysis between species and water treatments, and the limited September data set is used for correlation analysis with pore water chemistry described under “Correlations with carbon fluxes”. In this section you should just clearly state what data set is used when to stop the reader from making assumptions about your statistical design. Response: We agree this was confusing and edited to clarify. Text now reads “We used the September gas sampling campaign data to investigate correlations with other variables as it aligned temporally with when we collected biomass and pore water chemistry data.”

Lines 270-274: There is a lot of information in this sentence, can it be broken up into two separate sentences for improved readability. Response: We broke up into two sentences as recommended.

Line 577/78: The conclusion is not the place for this new reference to nanoparticles. Response: We altered text so that it is forward thinking and more appropriate for the conclusion; it now reads: “We recommend further examination of the interactions among emerging contaminants that can exacerbate water quality issues [71], vegetation, and wetland C cycling.”

Reviewer #2: I think this reviewed manuscript is a good improvement of the initial submission. Clarifying the choices about the statistical tests, along with a more clear methodology makes the paper stronger. I agree with the authors that discussing microbial function based on their diversity and abundance data may be overstepped. I like the new section “experimental design constrains” that the authors now provide, and that is central in the experimentation on analogue models
My last minor comments are:
- authors stated that their sampling was done away from the root influence. Their results may suggest otherwise, as for instance the Shannon’s diversity index significantly varied with the vegetation.

Response: Great point. We edited the discussion to address this. Lines 508-527 now read:

“Plant species played a significant, if small role in sediment microbial community composition in our study (Fig 5). The rhizosphere of wetland plants, the zone of soil directly in contact with the plant root has been shown to harbor elevated bacterial activity and altered communities in comparison to bulk soils [65,66]. While we did not specifically isolate rhizosphere soils, we observed differences in Shannon’s diversity among vegetation treatments in bulk soils, suggesting the pervasive influence of vegetation on soil bacteria. However, the majority of the identified bacterial OTUs were present in all three vegetation treatments in similar proportions, with only two OTU’s identified as significantly different in relative abundance among the three plant species (Fig 7). Thus, most bacteria appeared largely indifferent to plant species. In this regard, the influence of the plant on sediment microbial communities may be mostly limited to sediments in direct contact with roots. Instead, sediment microbial communities appeared to respond to changes in sediment properties, particularly those associated with salting, such as osmotic stress.”
- It is also unclear to me why the authors have made 3 samplings for CO2 uptake rates and CH4 emissions, when they did only one harvesting for the others.

Response: We quantified biomass and pore water chemistry at the peak of the growing season which is typical for plant ecology studies. Clearly it would have been ideal to have biomass and pore water chemistry data when we sampled carbon fluxes, but given personnel, time, and funding constraints, this was not possible.
- The authors haven’t addressed the questions about the (likely occurring, but not measured) pH and oxidative state changes that could be influenced by time, the different treatments and the experimental setup (draining before winter). It needs to be acknowledge somewhere that they could be factors explaining the biogeochemical change observations

Response: In the discussion we do mention the potential for alterations of redox conditions associated with winter mesocosm draining (Lines 571- 575)- “We manipulated the hydroperiod of our mesocosms to promote reduced soils during the growing season; while draining the tanks during winter to prevent tanks from cracking may have altered microbial composition and redox conditions, consistent manipulation of soils and hydrology allows us to draw inferences about responses to our vegetation and water quality treatments” We additionally added a sentence to address concerns related to pH and oxidative state (Lines 575- 577): “Further, though we did not measure soil pH or the oxidative state of our mesocosms, it is possible that our treatments altered these physio-chemical parameters [37,62] and indirectly effected the biogeochemical changes we observed.”
